# Risk factors for failure of revision total hip arthroplasty using a Kerboull-type acetabular reinforcement device

**DOI:** 10.1186/s12891-017-1741-8

**Published:** 2017-09-02

**Authors:** Shinya Hayashi, Takayuki Nishiyama, Shingo Hashimoto, Tomoyuki Matsumoto, Koji Takayama, Kazunari Ishida, Kotaro Nishida, Ryosuke Kuroda

**Affiliations:** 1 Department of Orthopaedic Surgery, Kobe University Graduate School of Medicine, 7-5-1 Kusunoki-cho, Chuo-ku, Kobe, 650-0017 Japan; 2 Department of Orthopaedic Surgery, Kakogawa City Hospital, Kakogawa, Japan; 3 Department of Orthopaedic Surgery, Kobe Kaisei Hospital, Kobe, Japan

**Keywords:** Revision total hip arthroplasty, KT plate, Allograft, Beta-tricalcium phosphate, Hydroxyapatite

## Abstract

**Background:**

The present study aimed to identify the risk factors associated with revision total hip arthroplasty (THA) failure using a Kerboull-type (KT) plate.

**Methods:**

We analyzed 77 revision THAs using cemented acetabular components with a KT plate for aseptic loosening between May 2000 and March 2012. We examined the association of bone graft type, acetabular bone defects, age at the time of surgery, preoperative Japanese Orthopaedic Association (JOA) score, postoperative JOA hip score, and body mass index, with radiographic failure as the outcome.

**Results:**

The 7.4-year radiographic failure survival rate was 81.6%. The survival rate was significantly different between the beta-tricalcium phosphate (β-TCP) group and the bulk allograft group (*p* = 0.019). The survival curves were also significantly different between the β-TCP group and bulk allograft group (*p* = 0.036). American Academy of Orthopaedic Surgeons type IV was significantly associated with radiographic failure (odds ratio [OR]: 15.5, 95% confidence interval [CI]: 1.4–175.4; *p* = 0.032).

**Conclusions:**

The midterm outcomes of revision THA indicate that type of bone graft and bone defect size may affect radiographic survival rate when using a KT plate.

## Background

Outcomes following THA have improved owing to advancements in prostheses and surgical procedures [1–3]. However, failure of acetabular components can lead to large bone defects with residual bone loss. It is well documented that bone defects determine outcome and management after revision hip surgery [4].

To compensate for large bone loss, prosthetic augmentation with bone grafting was developed, and several types of reinforcement devices are currently in use for revision THA, including the Burch-Schneider anti-protrusion cage [5], Mueller support ring [6], Ganz reinforcement ring [7], Kerboull device [8, 9], and Kerboull-type (KT) device [10, 11] (Table 1).

**Table 1:** Results of previous studies

Authors	Device type	Cases	Bone defect classification	Mean f/u years	Survival rate (loosening endpoint)
Ilyas et al. [5]	Burch-Schneider cage	33	AAOS III, IV	6.2y	85%
Schlegel et al. [6]	Mueller reinforcement ring	164	AAOS I-IV	8y	95%
Kerboull et al. [8]	Kerboull reinforcement device	53	AAOS III-IV	13y	92.1%
Kawanabe et al. [9]	Kerboull reinforcement device	42	AAOS II-IV	8.7y	67%
Baba et al. [10]	KT-plate	18	AAOS II, III	5y	87.5%
Hori et al. [11]	KT-plate	32	AAOS III, IV	10y	82.1%

**Fig. 1:**
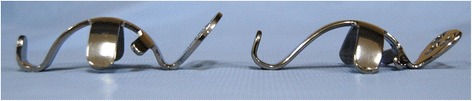
Photographs of the KT plate in the original position (*left*) and high placement position (*right*)

The use of a Kerboull reinforcement device can reduce excessive bone graft loading during incorporation and remodeling processes [12, 13]. The KT plate, a modified Kerboull plate made of titanium with a similar shape, has various offsets and vertical lengths (Fig. 1). Therefore, it is particularly useful for treating severe dysplasia or large bone defects of the acetabulum. Since 2000, we have used the KT plate with artificial bone materials or bulk grafts fashioned from a femoral head allograft.

Bone graft substitutes in combination with or without human bone are widely used for reconstruction of massive acetabular bone defects in revision surgery [13–16]. Good results have been associated with bone graft substitutes for acetabular bone defects [13–16] (Table 2).

**Table 2:** Results of previous studies

Authors	Type of graft	Cases	Bone defect classification	Mean f/u duration	Survival rate (loosening endpoint)
McNamara et al. [13]	allograft and hydroxyapatite	37	AAOS I-III	56.9m	95%
Oonishi et al. [14]	hydroxyapatite	40	AAOS I-II	4-10y	93%
Schwartz et al. [15]	biphasic calcium phosphate ceramics	29	Data not specified	more than 6m	100%
Blom et al. [16]	allograft and tricalcium phosphate-hydroxyapatite	43	Data not specified	24m	98%

Factors such as bone defect size, bone graft, age, and postoperative activity level may affect implant survival following revision THA, but no study has identified the critical risk factors for failure of revision THA using a KT plate. We hypothesized that factors specific to patient status or bone grafting may be predictive of poorer radiographic outcomes after revision THA. Therefore, the aim of this study was to evaluate the association between survival rates for radiological loosening and results according to bone defect or type of graft. Factors such as age and postoperative activity level were also considered.

## Clinical relevance

- Unlike prior reports, our study analyzed predictive factors for radiographic failure after revision THA.

## Methods

### Patients and surgery

A total of 95 consecutive revision THAs (86 patients) for aseptic loosening using cemented acetabular components with a KT acetabular reinforcement device (KT plate; KYOCERA Medical Corporation, Kyoto, Japan) were performed between May 2000 and March 2012. Exclusion criteria were recurrent dislocations and revision due to infection (8 cases); 10 cases were excluded because of loss to follow-up. Therefore, the data from 77 hips (6 men and 71 women) were included in the analysis. Initial diagnoses comprised osteoarthritis (59 hips), idiopathic osteonecrosis of the femoral head (4 hips), and rheumatoid arthritis (14 hips) (Fig. 1). Thirty-one patients underwent both acetabular- and femur-side revision surgery (cemented stem: 11 patients; cementless-stem: 20 patients), and 46 patients underwent only acetabular-side surgery (Fig. 3). The KT plate is available with an inside dome diameter of 44 mm, 48 mm, or 52 mm, and a vertical offset of +0 mm, +10 mm, or +15 mm. We used the 44-mm model in 20 patients, the 48-mm model in 35 patients, and the 52-mm model in 22 patients; we used the +0-mm vertical offset in 37 patients, the +10-mm vertical offset in 31 patients, and the +15-mm vertical offset in 9 patients. The inner head size diameter was 28 mm or 32 mm. We used an inner head size of 28 mm in 43 patients and 32 mm in 34 patients (Fig. 3).

**Fig. 2:**
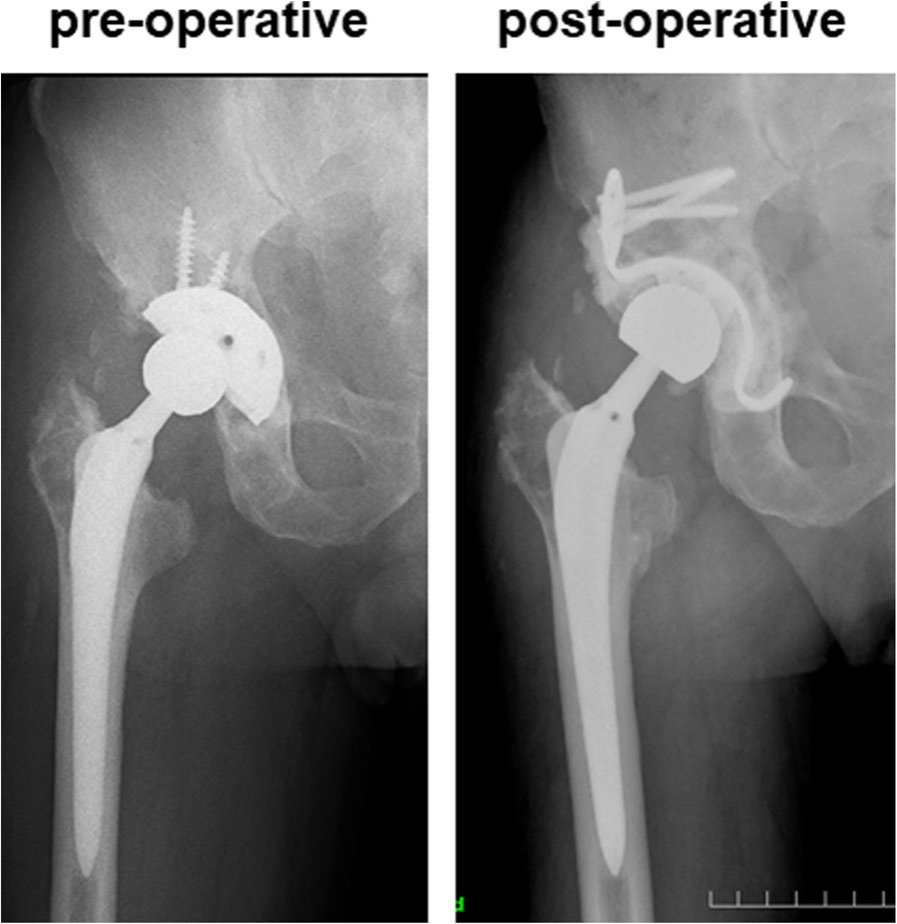
Representative case of preoperative and postoperative X-rays

Surgeries were performed using a direct lateral approach. All surgeries were performed by three experienced senior surgeons. The degree of acetabular bone defect was assessed after removal of the loosened implant, and the acetabular bone defect was augmented with a bone graft. The KT plate was fixed firmly by an inferior hook to the teardrop and a superior flange with at least two screws to the ilium. The polyethylene component was then cemented into the dome of the plate. When fixation of the inferior hook to the teardrop was not possible due to loss of host bone, the inferior hook of the KT plate was fixed to the ischium, inferior acetabular dome, or allograft. Between May 2000 and February 2003, bone defects were filled with β-TCP granules (OSferion; Olympus, Tokyo, Japan). Between February 2003 and March 2009, the bone defect was filled with HA block (Osteograft, Apaceram; KYOCERA Medical Corporation, Kyoto, Japan). Since 2009, we have used femoral head allografts that were harvested under sterile conditions and stored at −80 °C. After removing the remaining cartilage and soft tissue, the femoral head was cut using an oscillating saw into an appropriate shape and size to be used as a bulk allograft. A representative case with preoperative and postoperative X-rays is shown in Fig. 2. Postoperatively, full weight-bearing was tolerated by all patients within 3 days of surgery and patients were discharged from the hospital with a T-cane gait at 3 weeks postoperatively. The weight-bearing protocol was not changed over the course of the study period. All data were obtained in accordance with the World Medical Association Declaration of Helsinki Ethical Principles for Medical Research Involving Human Subjects.

### Postoperative evaluation

**Fig. 3:**
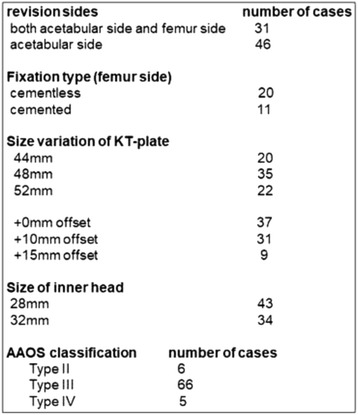
Summary of revision THA and number of patients according to AAOS classification. AAOS: American Academy of Orthopaedic Surgeons

Hip function was evaluated using the Japanese Orthopaedic Association (JOA) score, which allocates 40 points for pain, 20 points for range of motion, 20 points for walking ability, and 20 points for activities of daily living, with a maximum total score of 100 points [17]. The JOA score was evaluated preoperatively and at the final follow-up assessment. Body mass index (BMI) and University of California, Los Angeles (UCLA) activity scores were also evaluated at the final follow-up assessment. Acetabular defects were classified by one senior experienced surgeon according to the American Academy of Orthopedic Surgeons (AAOS) grading system [11]. Type II defects (cavitary bone loss) were found in 6 hips (7.8%), type III defects (cavitary and segmental bone loss) in 66 hips (85.7%), and type IV defects (pelvic discontinuity) in 5 hips (6.5%) (Fig. 3).

Radiological failure was defined by any of the following three criteria, according to Kawanabe [9]: 1. substantial migration defined as a change in the inclination angle of greater than 3° or migration greater than 2 to 3 mm; 2. progressive radiolucent line greater than 2 mm wide in all three zones as defined by DeLee and Charnley [18]; or 3. breakage of screws or device without migration or change in inclination. The postoperative and final follow-up radiographs were compared to assess radiological failure of the implant. The failure rate may be affected by the follow-up duration. Therefore, we performed survivorship analysis using radiographic failure as the endpoint.

### Statistical analysis

Demographic data were recorded as the mean ± standard deviation (SD) unless otherwise indicated. All data were normally distributed. Statistical analysis was performed using one-way analysis of variance with the Tukey post hoc test for multiple comparisons of paired samples. Cumulative probabilities of radiographic failure rate were estimated by using the Kaplan-Meier method. The survivorship curves for various subgroups were compared using the log-rank test. Logistic regression was performed to examine the association of age at the time of the operation, preoperative JOA score, postoperative JOA score, BMI, UCLA activity score, and AAOS classification with radiographic failure. Odds ratios (ORs) and 95% confidence interval (CIs) were calculated. Multivariate analysis was performed to adjust for potential confounders of age at the time of operation, preoperative JOA score, postoperative JOA score, BMI, UCLA activity score, and AAOS classification. The database was analyzed using SPSS software (IBM Corp., Armonk, NY, USA). Values of *p* < 0.05 were considered significant.

## Results

### Patient background

**Fig. 4:**
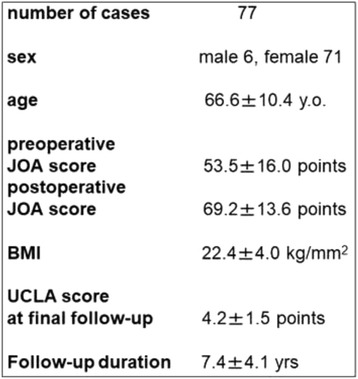
Patient demographic data. JOA: Japanese Orthopaedic Association, THA: total hip arthroplasty, BMI: body mass index, UCLA; University of California, Los Angeles

This study is a retrospective review of patient records. The patient background is shown in Fig. 4. The JOA score at the final follow-up examination increased significantly (*p* < 0.001). The patient background including pre- and postoperative JOA score, BMI, and UCLA score at final follow-up were not significantly changed according to the bone defect type or type of bone graft.

### Radiographic survival rate

**Fig. 5:**
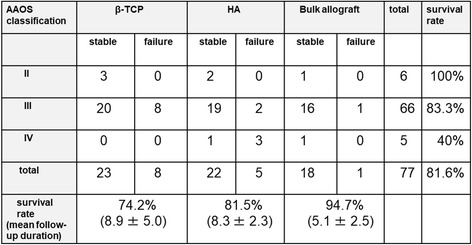
Number of radiographic failure cases according to type of bone graft and AAOS classification. AAOS: American Academy of Orthopaedic Surgeons, β-TCP: beta-tricalcium phosphate, HA: hydroxyapatite

Radiographic failure was evaluated for revision THA with β-TCP, hydroxyapatite (HA), and bulk allografts using cemented sockets with a KT plate. The total survival rate was 81.6%. The survival rate was 74.2% in the β-TCP group, 81.5% in the HA group, and 94.7% in the bulk allograft group (Fig. 5). However, the survival rate in the β-TCP group was significantly lower than in the HA (*p* = 0.048) or bulk allograft groups (*p* = 0.019). We also evaluated the stability of the acetabular component using the AAOS classification. The survival rate was 100% in the AAOS type II group, 83.3% in the type III group, and 40% in the type IV group (Fig. 5). The survival rate in the AAOS type IV group was significantly lower than in the type III group (*p* = 0.033).

**Fig. 6:**
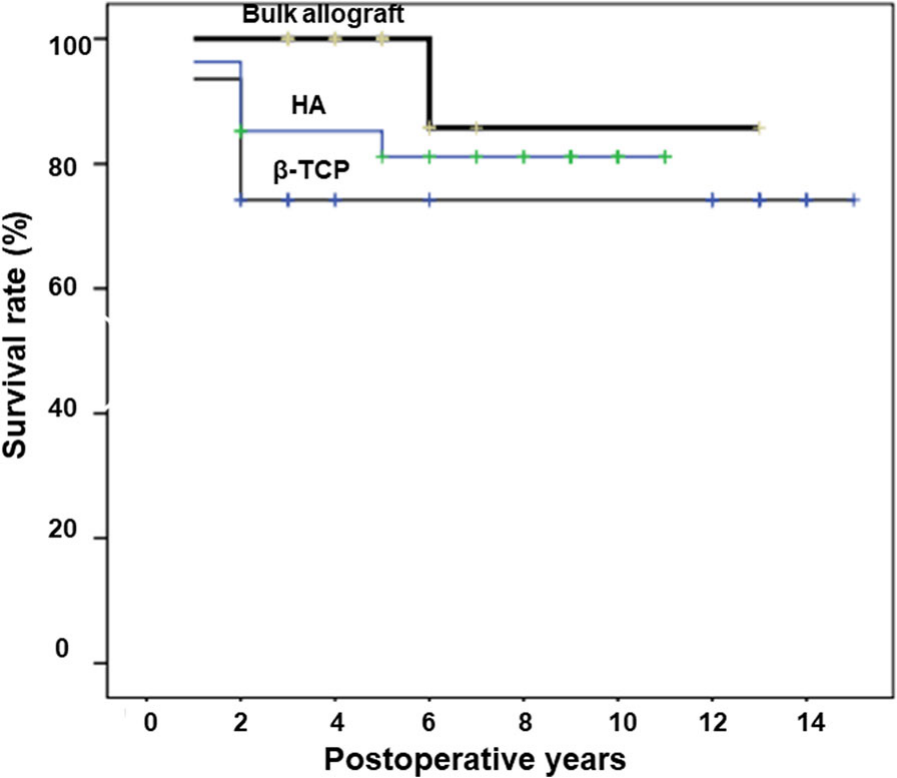
A Kaplan-Meier survival curve with radiographic failure as the endpoint. β-TCP: beta-tricalcium phosphate, HA: hydroxyapatite

We further performed survivorship analysis using radiographic failure as the endpoint. The survival curves were significantly different between the β-TCP group and bulk allograft group (*p* = 0.036), but were not significantly different between the HA group and bulk allograft group (Fig. 6).

#### Risk factors for radiographic failure

To identify risk factors for radiographic failure, we performed multivariate analysis to test the association of AAOS classification, clinical factors including age, pre- and postoperative JOA scores, BMI, UCLA activity score, and follow-up duration with radiographic failure and revision. AAOS type IV defect was found to be a risk factor for radiographic failure (OR: 15.5, 95% CI: 1.4–175.4, *p* = 0.032).

## Discussion

The core evaluation in this study focuses on the relationship between survival rates for radiological loosening and results according to bone defect or type of graft. We demonstrated that the 7.4-year survival rate was 81.6% when radiographic failure was the endpoint. These results are similar to those reported by previous studies [9–11].

Various types of graft have been used for reconstruction of acetabular bone defects. Blom et al. reported a 2-year survival rate of 100% in revision THA with impaction bone grafting of the acetabulum using a mix of bone chips and 80% TCP/20% HA [16]. In an in vitro study, Bolder reported that impaction bone grafting using TCP/HA and human bone chips significantly decreased socket migration in comparison with the use of human bone chips alone [19]. Aulakh et al. reported that the 13-year survival rate in revision THA with impaction bone grafting using a mix of 50% HA and 50% bone chips was 82%, and 84% with impaction bone grafting using only bone chips [20]. There was no significant difference between the two groups [20]. Tanaka et al. reported a 5-year survival rate of 100% in THA using a KT plate with HA granules [19].

Many good outcomes accompanied acetabular reconstructions of revision THA using artificial bone grafts [16, 19–21]. Kawanabe et al. compared the outcomes of morselized allografts and bulk allografts, and found the 10-year survival rate was 53% for morselized allografts and 82% for bulk grafts [9]. The authors also found the failure rate in the morselized allograft group was significantly higher than in the bulk allograft group when the acetabular defect was large (i.e., AAOS type III/IV) [9]. Based on our observations, the survival curves were significantly different between the β-TCP group and bulk allograft group. These results indicate the type of bone graft may affect radiographic survival rate when using a KT plate.

We further evaluated risk factors for failure of revision THA in association with clinical factors (age at the time of the operation, preoperative JOA score, postoperative JOA score, BMI at final follow-up assessment, follow-up duration, and UCLA activity score at final follow-up assessment), type of bone graft (β-TCP, HA, bulk allograft), and AAOS bone defect classifications. Only AAOS type IV was found to be a significant risk factor for failure of revision THA. Based on this finding, bone defect size is the critical risk factor for failure of revision THA using a KT plate.

Other reconstruction devices can fix the posterior wall in the case of massive host bone defect [20, 21]. Rogers et al. reported that an ilioischial cage or cup cage was able to fix the device to both the superior and inferior host bone with screws [20]. Friedrich et al. reported reconstruction for pelvic discontinuity using a patient-specific custom-made implant [21]. We are not aware of any reports of posterior plating combined with the use of a KT plate for pelvic discontinuity. Nonetheless, posterior plating might improve the survival rate of revision surgery with a KT plate in pelvic discontinuity.

A major limitation of this study is its small cohort size for statistical analysis. However, the number of hips evaluated in the study was quite high in comparison with other clinical studies of acetabular revision surgery [9–11, 19]. Another limitation is that three types of bone grafts were used at different times. Further, the mean follow-up duration was longer in the β-TCP group (mean: 8.9 ± 5.0 y) and HA group (mean: 8.3 ± 2.3 y) compared to the bulk allograft group (mean: 5.1 ± 2.5 y). These limitations may affect the survival result.

## Conclusion

The midterm outcomes of revision THA indicate that type of bone graft and bone defect size may affect radiographic survival rate when using a KT plate. New devices and techniques for KT plates are needed to improve the treatment of pelvic discontinuity.

## Abbreviations

β-TCP: beta-tricalcium phosphate
AAOS: American Academy of Orthopaedic Surgeons
BMI: Body mass index
HA: Hydroxyapatite
JOA: Japanese Orthopaedic Association
OR: Odds ratio
THA: Total hip arthroplasty
UCLA: University of California, Los Angeles
